# Molecular characterization of the heterogeneity of satellite cell populations isolated from an individual Turkey pectoralis major muscle

**DOI:** 10.3389/fphys.2025.1547188

**Published:** 2025-02-20

**Authors:** Hui Yu, Zhenyang Li, Joseph Yimiletey, Chunmei Wan, Sandra Velleman

**Affiliations:** Department of Animal Sciences, The Ohio State University, Columbus, OH, United States

**Keywords:** satellite cells, heterogeneity, Turkey, pectoralis major muscle, RNAsequencing

## Abstract

Satellite cells (SCs) are myogenic stem cells responsible for post hatch muscle growth and the regeneration of muscle fibers. Satellite cells are not a homogenous population of cells within a muscle and have variable rates of proliferation and differentiation even within a single fiber type muscle like the turkey pectoralis major muscle. In this study, the single satellite cell clones derived from the same turkey pectoralis major muscle with different proliferation rates were compared. The clones were classified as either fast-growing (early clone) or slow-growing (late clone) SCs. To thoroughly examine the molecular differences between these two groups, RNA sequencing was conducted to compare their transcriptomes following 72 h of proliferation. Principal Component Analysis confirmed that the transcriptomic profiles of early- and late-clones are markedly distinct. Differential gene expression analysis identified over 5,300 genes that were significantly differentially expressed between the two groups of cells. Gene ontology analysis showed that genes highly expressed in early clones are responsible for the fundamental aspects of muscle biology, including muscle tissue development and structural maturation. Conversely, genes upregulated in late clones are involved in cell-cell communication, extracellular matrix interactions, signal ligand activity, and cytokine activity—key components for forming an extracellular niche essential for functional satellite cells maintenance. Further examination of specific gene ontology categories such as muscle structure development and extracellular matrix components indicated significant differences in gene expression patterns between early- and late-clones. These findings highlight the genetic and functional diversity of SCs in turkeys. The distinct roles of these satellite cell populations indicate that a balance between them is necessary for preserving the normal physiological functions of SCs.

## 1 Introduction

In food animal agriculture, it is crucial to identify mechanisms that can enhance the efficiency of muscle growth to meet human nutritional needs. In the United States, poultry is the most consumed type of meat, with consumption rates steadily increasing. Turkey meat, valued at approximately $6.57 billion, accounts for 10% of the poultry meat revenue, according to the United States Department of Agriculture (Poultry - Production and Value 2023 Summary) (NASS, 2023). Maintaining sustainability in our food supply chains is necessary for ensuring continuous food availability and improving the health and wellbeing of current and future populations. Sustainable production of animal protein is vital for meeting meat consumption demands (Duggan et al., 2023). In the commercial turkey industry, the breast muscle (pectoralis major muscle; p. major) is the most economically valuable muscle. Efficient development of the *p. major* muscle remains a primary focus in turkey breeding strategies.

In poultry, the mechanism that drive skeletal muscle development differ between the embryonic and post-hatch stages. During the embryonic stage, skeletal muscle develops through the proliferation and differentiation of myoblasts, which fuse to develop multinucleated myotubes. These myotubes then differentiate into mature muscle fibers, producing muscle-specific contractile proteins. Myofiber formation is complete at hatch (Smith, 1963), and further growth occurs through the addition of nuclei to existing fibers from satellite cells (SCs), the adult myoblasts, leading to the enlargement of fibers via hypertrophic growth (Moss and Leblond, 1971; Cardasis and Cooper, 1975). Once growth is complete, SCs enter a quiescent state (Schultz et al., 1978), remaining in their niche until they are activated to repair and regenerate damaged muscle fibers (Yin et al., 2013).

Initially identified by Mauro (1961), SCs are located between the sarcolemma and the basal lamina of myofibers and comprise a heterogenous population of muscle stem cells (Schultz, 1974; Biressi and Rando, 2010; Tierney and Sacco, 2016). Khodabukus and Baar (Khodabukus and Baar, 2015) observed that SCs originating from slow and fast myofibers express corresponding slow and fast contractile proteins and retain the metabolic characteristics of their original myofibers. Even within the same regenerating muscle fiber, SCs divide asymmetrically, producing two distinct daughter cells—one that retains the capacity for stem cell self-renewal and another that commits to proliferation and differentiation (Kuang et al., 2007; Ono et al., 2010). SCs isolated from turkey lines with different growth rates show distinct responses to thermal stress and exhibit altered transcriptomic profiles (Reed et al., 2022a; Reed et al., 2022b). Notably, selective breeding for enhanced growth and increased breast muscle yield in turkeys has transformed the satellite cell (SC) population in the p.major muscle into cells with higher proliferation and differentiation rates, along with an elevated adipogenic potential (Clark et al., 2017; Velleman and Coy, 2020; Xu et al., 2021b).

The turkey p.major muscle consists homogeneous Type IIb fibers; however, even within this single fiber-type muscle, McFarland et al. (1995). identified significant heterogeneity among SCs. In this study, 73 distinct SCs were isolated from the *p. major* muscle of a 6-week-old Nicholas tom turkey. Each single-cell clone was cultured, expanded, and categorized based on its proliferation rate. The fastest-growing cells reached 65% confluency within 17 days (early clones), whereas the slowest-growing cells required 30 days to reach the same confluency level (slow clones), highlighting substantial variability in growth rates among the isolated cells. Subsequent biological analyses revealed that the early clones exhibited greater responsiveness to fibroblast growth factor 2 stimulation and expressed higher levels of *fibroblast growth factor receptor 1* at the onset of proliferation. During differentiation, these cells also showed elevated production of heparan sulfate proteoglycan (McFarland et al., 2003). Furthermore, a follow-up study demonstrated that the early clones were more sensitive to the inhibitory effects of transforming growth factor beta on both proliferation and differentiation compared to the slower-growing late cells (Yun et al., 1997). Based on these previous findings, we hypothesized that early and late clones represent two genetically distinct populations characterized by fundamentally different transcriptomic profiles. These intrinsic genetic differences likely drive their varied responses to biological stimuli, influencing key processes such as proliferation, differentiation, and responsiveness to growth factors and signaling molecules. To test this hypothesis, we compared the transcriptional differences between the early and late clones after 72 h proliferation. Gene functional analysis was conducted to characterize the key molecular pathways and mechanisms that differentiate the 2 cell populations. Uncovering the satellite cell-mediated mechanisms involved in the development of the *p. major* muscle will facilitate the development of strategies to promote animal growth and meat production.

## 2 Materials and methods

### 2.1 Turkey myogenic satellite cells

The satellite cell clones utilized in the current study were previously developed by McFarland et al. (1995). Briefly, SCs were isolated from the pectoralis major muscle of a 6-week-old Nicholas tom turkey, sourced from a local producer raising them for consumption purposes. These SCs were suspended in McCoy’s 5A medium, and individual cells were selected and transferred to a 96-well cell culture plate using the Quixell cell manipulator robotic system (Stoelting Co., Wood Dale, IL). This instrument utilizes a micropipette to isolate individual suspended SCs and place them into wells to growth cell clones. These clones were categorized based on their proliferation rates. Clones that reached confluency within 17–19 days in a 25 cm^2^ tissue culture flask were considered as early clones, reflecting their fast growth/proliferation rates. In contrast, clones requiring 28–29 days to achieve confluency were classified as slow clones, indicative of slower growth/proliferation rates. All clones were preserved in liquid nitrogen for future use.

The early and late clones were cultured as described previously (Reed et al., 2017; Xu et al., 2021b). Briefly, the same number of cells were plated and incubated at 38°C for 24 h in plating medium consisting of Dulbecco’s Modified Eagle’s Medium (D5523, Sigma Aldrich, St. Louis, MO), 10% chicken serum (C5405, Sigma Aldrich, St. Louis, MO), 5% horse serum (H1270, Sigma Aldrich, St. Louis, MO), 1% antibiotics-antimycotics (30004CI, Corning, New York, NY), and 0.1% gentamicin (GT-10, Omega Scientific, Tarzana, CA). After 24 h, the cells were changed to a feeding medium consisting of McCoy’s 5A Medium (M4892, Sigma Aldrich, St. Louis, MO), 10% chicken serum, 5% horse serum, 1% antibiotics-antimycotics, and 0.1% gentamicin, and cultured at 38°C for an additional 72 h. The growth medium was refreshed every 24 h during this 72-h proliferation period. At harvest, the cell medium was removed, and the cells were washed twice with phosphate-buffered saline before being collected into TRIzol Reagent (15596018, ThermoFisher, Waltham, WA) and stored at −80°C until RNA isolation.

### 2.2 RNA isolation and sequencing

Total RNA was isolated from each sample using TRIzol Reagent, following the manufacturer’s instructions. RNA integrity and quantification were assessed at The Genomics Shared Resource, The Ohio State University, using the Agilent 2,100 Bioanalyzer. Only RNA samples with an RNA Integrity Number (RIN) greater than 9 were sent to Innomics Inc. (Sunnyvale, CA). for sequencing. Sequencing was performed on the DNBSEQ platform with stranded paired-end 150 bp reads. Four replicates were included for each cell line.

### 2.3 RNAseq data analysis

Quality control checks on raw sequence data from each sample were performed with FastQC (v0.12.1) (Andrews, 2010). Reads were aligned to reference turkey genome (UMD 5.1, ENSEMBL Annotation 111) using STAR (v2.7.11b) with default parameters (Dobin et al., 2013). All samples passed the post-alignment quality check (QualiMap v.2.3) (Okonechnikov et al., 2016). The DEseq2 (Love et al., 2014) method was used for differential expression analysis comparing early and late clones. The adjusted p-value was calculated using the default “BH” setting in DESeq2, which controls the false discovery rate (FDR)—the expected proportion of false discoveries among the rejected hypotheses. The FDR is a less stringent condition than the family-wise error rate, making these methods more powerful compared to others (Benjamini and Hochberg, 1995).

Gene identifiers for annotated genes were obtained through an iterative process from multiple sources. Primarily, these identifiers were sourced directly from the ENSEMBL annotations. For genes lacking corresponding ENSEMBL IDs, annotations were obtained from Dr. Kent M. Reed and relevant publications from his research group (Reed et al., 2017; Barnes et al., 2019; Reed et al., 2022a; Reed et al., 2022b). Gene ontology analysis was performed using g: Profiler (available at https://biit.cs.ut.ee/gprofiler/gost) (Kolberg et al., 2023). The input consisted of genes with a log fold change (logFC) greater than 2 and a *P <* 0.01. *Meleagris gallopavo* (Turkey) was selected as the reference genome for the analysis. The g: SCS algorithm was used for computing multiple testing correction for p-values gained from GO and pathway enrichment analysis. The top five Gene Ontology (GO) terms were presented in Figure 3. The specific gene ontology terms related to muscle structure development, extracellular matrix, signaling receptor activator activity, and cytoskeletal protein binding were retrieved from EBI QuickGO database (https://www.ebi.ac.uk/QuickGO/). The PANTHER Overrepresentation Test (Protein Analysis Through Evolutionary Relationships, Version 19.0, released on 2024–06–20) was conducted using the PANTHER database (https://pantherdb.org/). *Gallus gallu*s was selected as the reference organism and reference gene list. The analysis employed Fisher’s Exact Test with Bonferroni correction for multiple testing. The top five GO terms identified are presented in Tables 1, 2. The input genes were selected based on a Log_2_FC greater than 2 and a *P <* 0.05.

## 3 Results

### 3.1 Summary of overall gene expression

Total RNA was extracted from both early clones (n = 4) and late clones (n = 4), with each sample comprising pooled material from 3 wells to construct individual barcoded libraries. Sequencing generated over 257 million 150 bp paired-end reads (SRA BioProject: PRJNA1196520). The number of reads per library ranged from 28.6 to 35.3 million, with an average of 32.2 million (Table 1). The average proportion of nucleotides with a quality score above 20 (Q20) was over 98.5%, and those with a quality score above 30 (Q30) averaged 95.5%. The results from replicate libraries were consistent.

**TABLE 1:** Summary of RNAseq data for 72 h proliferation experiment. For each library the total number of raw reads, Q20 (%), Q30 (%), the number of observed genes (mapped reads >1) by library and the percentage of uniquely mapped reads are given.

Cell line	Replicates	PE reads	Q20 (%)	Q30 (%)	GC content (%)	Observed genes	Uniquely mapped reads %
Early Clones	1	28,649,147	98.74	96.53	48.51	13,431	82.11%
	2	28,867,578	98.77	96.62	48.35	13,500	83.28%
	3	32,174,596	98.67	96.26	48.27	13,679	83.42%
	4	35,349,407	98.81	96.71	48.30	13,712	83.35%
Late Clones	1	34,603,174	98.74	96.50	48.03	13,611	86.44%
	2	33,454,390	98.69	96.39	48.19	13,545	86.35%
	3	33,739,352	98.74	96.49	48.14	13,541	86.03%
	4	30,894,176	98.74	96.55	48.95	13,497	84.48%

**FIGURE 1:**
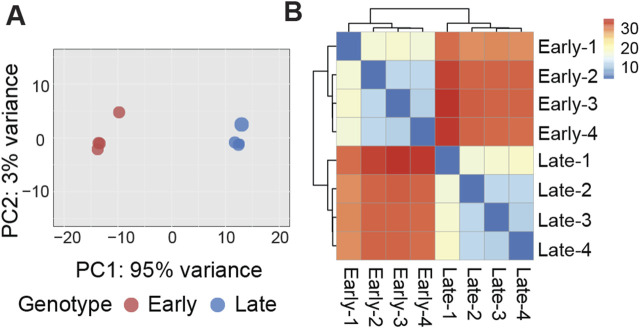
**(A)** Graphical representation of the first (PC1) and second (PC2) principal components (PC) affecting gene expression patterns in early and late cell clones. **(B)** Heatmap of sample-to-sample distance matrix showing the dissimilarities between early clones and late clones and the similarity between samples within each clone.

Evidence of expression (at least one mapped read per library) was observed for an average of 13,565 genes, with a nearly equal distribution between early clones (13,581) and late clones (13,549). Over 82% of the reads uniquely mapped to the turkey genome (Table 1). We conducted Bartlett’s test of sphericity, with a result of *p* < 0.0001, and the Kaiser-Meyer-Olkin (KMO) test, with an overall KMO of 0.91. These results suggest that the data is suitable for factor analysis. Subsequently, we carried out principal component analysis (PCA), which revealed distinct clustering of early and late clones along the first two principal components based on normalized read counts, as visualized in Figure 1A. This analysis highlights the substantial variations between the 2 cell populations. Technical replicates clustered closely as nearest neighbors within the PCA space, confirming the validity of pooling replicates for expression analysis. Heatmaps, organized by early and late clones, revealed a distinct separation between the two groups while preserving the relationships within each group of cells (Figure 1B), further supporting the significant differences between early and late clones.

### 3.2 Differential gene expression

**FIGURE 2:**
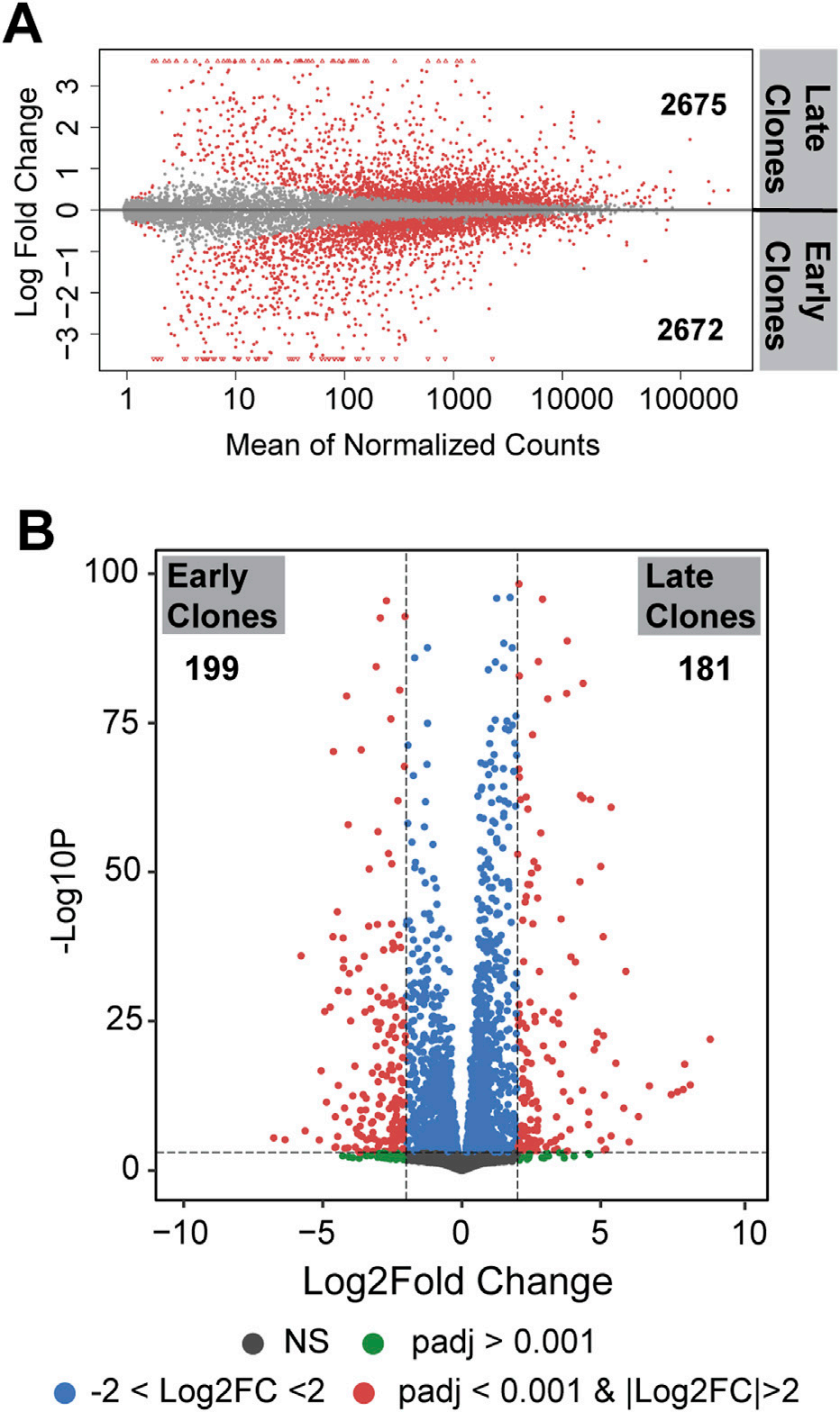
**(A)** MA plot (Bland-Altman plot) depicting differentially expressed genes on a logarithmic scale. Genes with *p* < 0.05 are labeled in red. The number indicated the upregulated genes in each clones. **(B)** Volcano plot presenting differentially expressed genes. Genes with *p* < 0.001 and |Log_2_FC| >2 are labeled in red.

Differences in gene expression between the 2 cell populations are illustrated by the distribution of unique and shared expressed genes, as shown in the Bland-Altman plot (MA plot) in Figure 2A. The MA plot displays the log ratio (M) of gene expression against the average expression (A) to visualize differences between the two groups. A total of 5,347 genes were identified as differentially expressed genes (DEGs) with an adjusted *p* < 0.05; of these, 2,675 genes were upregulated in late clones and 2,672 genes were upregulated in early clones. A more detailed analysis of these DEGs, using a stringent fold change threshold of |Log_2_FC| >2 and an adjusted *p* < 0.001, identified 181 genes upregulated in late clones and 199 genes upregulated in early clones. This finding highlights the substantial differences between early and late clones, despite both exhibiting a similar number of differentially expressed genes (Figure 2B).

### 3.3 Gene functional analysis

Gene functional enrichment analysis was conducted on differentially expressed genes using two resources: the PATHER (Protein Analysis Through Evolutionary Relationships) knowledgebase and g:Profiler (g: GOSt Functional Profiling). The PATHER overrepresentation test applied genes mapped to chicken (*Gallus gallus*) gene sets, while g:Profiler analysis used turkey (*M. gallopavo*) as the input organism. The use of both resources ensured that the functional analysis results were validated against orthologous chicken gene sets, providing consistency and robust validation of the findings. For the analysis, DEGs with a fold change threshold of |Log_2_FC| >2 and an adjusted *p* < 0.05 were included in the PATHER analysis, while a more stringent adjusted *p* < 0.01 with the same fold change threshold was applied in the g:Profiler analysis.

**FIGURE 3:**
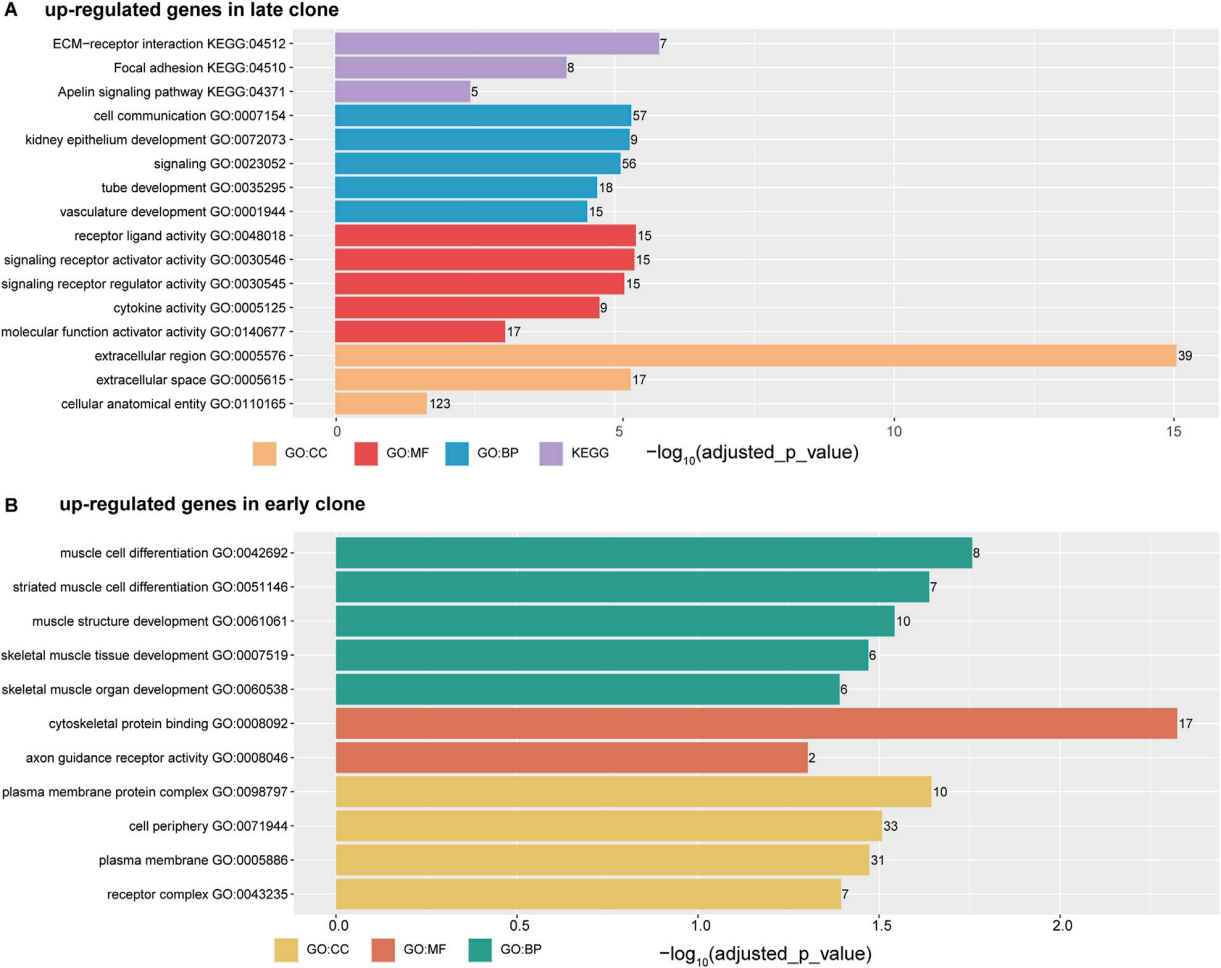
The horizontal bar chart illustrates the top five ontology terms, categorized into CC (Cellular Components), MF (Molecular Functions), BP (Biological Processes), and KEGG (Kyoto Encyclopedia of Genes and Genomes) pathways. The x-axis represents the -log_10_ (adjusted *p*-value), while the numbers displayed on each bar indicate the number of genes associated with each term. **(A)** Functional analysis of genes upregulated in late clones. **(B)** Functional analysis of genes upregulated in early clones.

The analysis of upregulated genes in late clones using g:Profiler (Figure 3A) revealed significant enrichment across various categories. In the GO Molecular Functions category, the most significant enrichments were observed for cell receptor ligand activity (-log_10_ (adjusted *p*-value) = 5.38), signaling receptor activator activity (-log_10_ (adjusted *p*-value) = 5.35), cytokine activity (-log_10_ (adjusted *p*-value) = 4.72), and molecular function activator activity (-log_10_ (adjusted *p*-value) = 3.04). For the GO Cellular Components category, the highest enrichment scores were found for the extracellular region (-log_10_ (adjusted *p*-value) = 15.04), extracellular space (-log_10_ (adjusted *p*-value) = 5.28), and cellular anatomical entity (-log_10_ (adjusted *p*-value) = 1.64). These findings align with results from the KEGG pathways, where extracellular-receptor interaction featured prominently (-log_10_ (adjusted *p*-value) = 5.79). In the GO Biological Processes category, notable enrichments were linked to functions such as cell communication (-log_10_ (adjusted *p*-value) = 5.29), signaling (-log_10_ (adjusted *p*-value) = 5.10), tube development (-log_10_ (adjusted *p*-value) = 4.68), and vasculature development (-log_10_ (adjusted *p*-value) = 4.50).

In contrast to the gene enrichment profile observed in late clones, the early clones exhibit a distinct pattern of enrichment (Figure 3B). The top enrichments in the GO Biological Processes category are primarily associated with muscle cell function and development, including muscle cell differentiation (-log_10_ (adjusted *p*-value) = 1.76), muscle structure development (-log_10_ (adjusted *p*-value) = 1.54), and skeletal muscle organ development (-log_10_ (adjusted *p*-value) = 1.47). In the GO Molecular Function category, notable enrichments include cytoskeletal protein binding (-log_10_ (adjusted *p*-value) = 2.32) and axon guidance receptor activity (-log_10_ (adjusted *p*-value) = 1.30). Meanwhile, the GO Cellular Components category shows significant enrichment in cell periphery (-log_10_ (adjusted *p*-value) = 1.51), plasma membrane (-log_10_ (adjusted *p*-value) = 1.47), and receptor complex (-log_10_ (adjusted *p*-value) = 1.39).

The examination of upregulated genes in late clones (146 IDs mapped to the *G. gallus* gene set, Table 2) using PATHER revealed significant enrichments in the GO Molecular Function category. The top enrichments included extracellular matrix structural constituent conferring tensile strength (27.33-fold, *p* = 9.13E-06), extracellular matrix structural constituent (17.49-fold, *p* = 3.08E-05), growth factor activity (12.58-fold, *p* = 6.56E-08), integrin binding (12.34-fold, *p* = 2.92E-03), and cytokine activity (8.97-fold, *p* = 8.06E-05). Within the GO Biological Processes category, extracellular matrix organization (9.70-fold, *p* = 8.30E-05) and skeletal system development (6.25-fold, *p* = 2.17E-02) emerged as the top two most highly expressed terms. For the GO Cellular Components category, significant enrichments were observed in the basement membrane (11.93-fold, *p* = 1.07E-02) and extracellular space (5.36-fold, *p* = 3.79E-16).

**TABLE 2:** PANTHER Overrepresentation test of upregulated DEGs in the late clones after 72 h of proliferation. Shown are the gene ontology categories with the greatest fold enrichment in the GO biological process category.

Upregulated gene ontology terms in late clones					
	*Gallus gallus* (18366)	Turkey DEG (146 out of 168)	Expected	Fold enrichment	*p*-value
GO molecular function					
Extracellular matrix structural constituent conferring tensile strength (GO:0030020)	28	7	0.26	27.33	0.000
Extracellular matrix structural constituent (GO:0005201)	50	8	0.46	17.49	0.000
Growth factor activity (GO:0008083)	113	13	1.03	12.58	0.000
Integrin binding (GO:0005178)	62	7	0.57	12.34	0.003
Cytokine activity (GO:0005125)	134	11	1.23	8.97	0.000
GO Biological Process					
Extracellular matrix organization (GO:0030198)	124	11	1.13	9.70	0.000
Skeletal system development (GO:0001501)	175	10	1.60	6.25	0.022
Cell adhesion (GO:0007155)	474	19	4.34	4.38	0.000
Animal organ development (GO:0048513)	856	27	7.83	3.45	0.000
Cell surface receptor signaling pathway (GO:0007166)	1,027	28	9.39	2.98	0.001
GO Cellular Component					
Basement membrane (GO:0005604)	55	6	0.50	11.93	0.011
Extracellular space (GO:0005615)	831	41	7.65	5.36	0.000

The analysis of upregulated genes in early clones (133 IDs mapped to the *G. gallus* gene set, Table 3) using PATHER revealed a different pattern compared to late clones. In the GO Cellular Components category, the top enrichments were associated with muscle structure, specifically myofilament (26.33-fold, *p* = 1.36E-02) and sarcomere (8.13-fold, *p* = 2.50E-02). These findings align with the muscle development functions identified in the g:Profiler analysis (Figure 3B). However, the GO Molecular Function and Biological Process categories in PATHER highlighted a unique emphasis on nervous system establishment. In the GO Molecular Function category, the top enrichments included axon guidance receptor activity (71.09-fold, *p* = 1.12E-02) and acetylcholine-gated monoatomic cation-selective channel activity (25.39-fold, *p* = 2.03E-04). Similarly, in the GO Biological Processes category, the terms with the highest expression were excitatory postsynaptic potential (18.85-fold, *p* = 3.48E-04) and chemical synaptic transmission (18.03-fold, *p* = 4.79E-04).

**TABLE 3:** PANTHER Overrepresentation test of upregulated DEGs in the early clones after 72 h of proliferation. Shown are the gene ontology categories with the greatest fold enrichment in the GO biological process category.

Upregulated gene ontology terms in early clones					
	*Gallus gallus* (18366)	Turkey DEG (133 out of 155)	Expected	Fold enrichment	*p*-value
GO molecular function					
Axon guidance receptor activity (GO:0008046)	5	3	0.04	71.09	0.011
Acetylcholine-gated monoatomic cation-selective channel activity (GO:0022848)	28	6	0.24	25.39	0.000
Semaphorin receptor binding (GO:0030215)	25	5	0.21	23.70	0.004
Transmitter-gated monoatomic ion channel activity involved in regulation of postsynaptic membrane potential (GO: 1904315)	49	8	0.41	19.35	0.000
Excitatory extracellular ligand-gated monoatomic ion channel activity (GO:0005231)	43	6	0.36	16.53	0.003
GO biological process					
Excitatory postsynaptic potential (GO:0060079)	44	7	0.37	18.85	0.000
Chemical synaptic transmission, postsynaptic (GO:0099565)	46	7	0.39	18.03	0.000
Neural crest cell development (GO:0014032)	54	6	0.46	13.17	0.027
Regulation of postsynaptic membrane potential (GO:0060078)	65	7	0.55	12.76	0.005
Stem cell development (GO:0048864)	56	6	0.47	12.70	0.033
GO cellular component					
Myofilament (GO:0036379)	18	4	0.15	26.33	0.014
Sarcomere (GO:0030017)	102	7	0.86	8.13	0.025
Postsynaptic membrane (GO:0045211)	156	9	1.32	6.84	0.007
Plasma membrane region (GO:0098590)	511	16	4.31	3.71	0.007
Neuron projection (GO:0043005)	620	17	5.23	3.252	0.020

Given the significant differences observed in the functional annotation of DEGs between early and late clones, we conducted a detailed analysis of individual gene ontology terms to further validate our findings. Using results from g:Profiler, we focused on gene ontology categories associated with upregulated genes in late clones, including the extracellular matrix (GO:0031012) and signaling receptor pathway (GO:0030546). Among the 1,502 genes associated with the extracellular matrix, 291 were identified in our dataset, and their expression patterns, as visualized in a heatmap, revealed clear distinctions between early and late clones (Supplementary Figure S1A). Similarly, of the 1,530 genes related to the signaling receptor pathway, 163 were mapped in our dataset, with their heatmap also showing distinct patterns between the two groups (Supplementary Figure S1B). In parallel, we examined two gene ontology terms associated with upregulated genes in early clones: muscle structure development (GO:0061061) and cytoskeletal protein binding (GO:008092). From the muscle structure development category, 229 out of 1,793 genes were detected in our dataset. The corresponding heatmap (Supplementary Figure S2A) highlighted distinct expression differences between early and late clones (Supplementary Figure S2A). Likewise, of the 2,484 genes associated with cytoskeletal protein binding, 665 were mapped in our dataset. The heatmap for these genes (Supplementary Figure S2B) further highlighted the divergent expression patterns between the two clones.

### 3.4 Top expressed genes in the 2 cell populations

**FIGURE 4:**
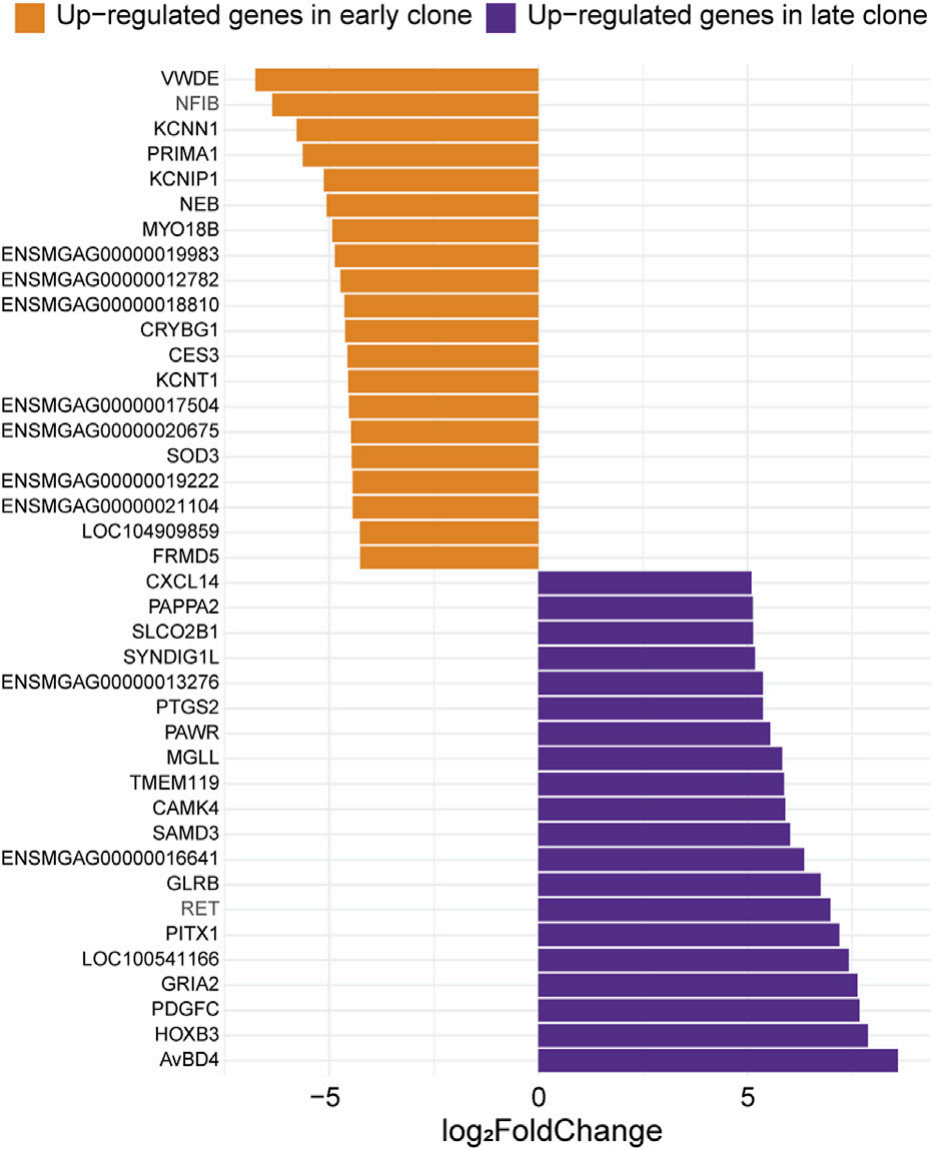
Expression levels of the top 20 genes with the most significant differences between early and late clones. The Log_2_FC for each gene is shown (adjusted *p*-value <0.05).

The 40 most significant differentially expressed genes with the greatest expression differences between the 2 cell populations are presented in Figure 4. Among these, the most highly upregulated gene in late clones (Log_2_FC = 8.60) was identified as an ortholog of *avian beta-defensin 4* (*AvBD4*). In chickens, this gene is part of a 14-member family of antimicrobial peptides known for their broad-spectrum activity and critical role in the innate immune system (Sugiarto and Yu, 2004). Also upregulated was *homeobox B3* (*HOXB3,* Log_2_FC = 7.88), a member of the HOX gene family, which is linked to embryonic development, as well as the progression of diseases and cancers (Chan et al., 2005; Alharbi et al., 2013; Chen et al., 2013). Another upregulated gene, *Platelet-Derived Growth Factor C* (*PDGFC,* Log_2_FC = 7.68), plays a key role in tissue remodeling, angiogenesis, and embryonic development (Reigstad et al., 2005). The *Prostaglandin Endoperoxide Synthase 2*, also known as *Cyclooxygenase 2* (*PTGS2/COX2,* Log_2_FC = 5.37), plays a crucial role in inflammatory signaling pathologies. Its induction leads to the production of Prostaglandin E2, essential for effective skeletal muscle stem cell function, enhancing regeneration and muscle strength (Ho et al., 2017; Martin-Vazquez et al., 2023).

The top upregulated gene in early clones is an ortholog of *Von Willebrand Factor D and EGF Domain-Containing Protein* (*VWDE*, Log_2_FC = −6.77), which is predicted to facilitate signaling receptor binding activity and play a role in anatomical structure development (Alliance of Genome Resources, 2024). Additionally, several upregulated genes in early clones are linked to neuronal excitability and synaptic function. These include *Potassium Calcium-Activated Channel Subfamily N Member 1* (*KCNN1*, Log_2_FC = −5.78), *Potassium Voltage-Gated Channel Interacting Protein 1* (*KCNIP1*, Log_2_FC = −5.12), and *Potassium Sodium-Activated Channel Subfamily T Member 1* (*KCNT1*, Log_2_FC = −4.55). The upregulation of the muscle-specific gene *Myosin 18B* (*MYO18B*, Log_2_FC = −4.93) suggests a role for early clones in sarcomere integrity and assembly (Berger et al., 2017). Furthermore, the association of *MYO18B* with certain myopathies highlights the potential involvement of early clones in both normal muscle function and disease (Alazami et al., 2015; Malfatti et al., 2015).

### 3.5 Expression of genes associated with muscle growth and SC function

Since the two SC populations were categorized by distinct cell proliferation rates, and McFarland et al. (2003) reported that early clones are more responsive to fibroblast growth factor 2 (FGF2), showing higher gene expression levels of *FGF2* and *FGF receptor-1* (*FGFR1*) at the onset of proliferation compared to slower-growing cells, we specifically examined the expression of *FGF2* and *FGFR1*. While there was no significant difference in *FGF2* expression (Log_2_FC = 0.12), fast-growing SCs exhibited marked higher expression of *FGFR1* (Log_2_FC = −1.74), consistent with the earlier report. Additionally, we analyzed the expression of other FGF family genes, including *FGF10, FGF12, FGF13, FGF16, FGF19,* and *FGF22*, and found no significant differences between the 2 cell populations. Intriguingly, *FGFBP1*, a secreted molecule that functions to chaperone FGF ligands from the extracellular matrix to cognate receptors—thereby enhancing the biological activity of FGF ligands—was significantly elevated in slow-growing SCs (Log_2_FC = 4.45), suggesting a possible alternative pathway in slow-growing cells to mediate FGF signaling (Abuharbeid et al., 2006; Briones et al., 2006).

In addition to the FGF gene family, we also explored the expression of receptors associated with insulin-like growth factors (IGFs), including the IGF1 receptor (IGF1R) and the IGF-binding protein (IGFBP) family. IGFs are known for their diverse roles as endocrine, paracrine, and autocrine factors that are crucial for cell growth, proliferation, differentiation, and survival (Allard and Duan, 2018). Although the changes were statistically significant, late clones exhibited an increase in *IGF1R* expression (Log_2_FC = 0.29) and a more pronounced upregulation of *IGFBP5* (Log_2_FC = 1.69). Notably, overexpression of *Igfbp5* has been shown to induce delayed muscle development in mice (Salih et al., 2004), suggesting that the significant elevation of *IGFBP5* in slow-growing SCs may play a role in their reduced proliferation rates.

Notch signaling is crucial for regulating SC functions such as proliferation, differentiation, and self-renewal. Activation of HES1 through this pathway modulates the transcription of the myogenic transcription factor *MYOD1* and the Notch ligand *DLL1*, thereby modulating the activation state of SCs (Gioftsidi et al., 2022; Vargas-Franco et al., 2022). Our analysis of key genes involved in Notch signaling revealed increased levels of *HES1* (Log_2_FC = −2.19), *MYOD1* (Log_2_FC = −0.64), and *DLL1* (Log_2_FC = −2.10) in fast-growing SCs, indicating constitutive activation of the Notch signaling pathway in these cells. Additionally, an elevated expression of the *MYOG* (myogenin, Log_2_FC = −1.51) gene, one of the key factors regulating myogenesis, was observed in the fast-growing SCs. However, the expression of the transcription factor *PAX7* (Log_2_FC = 0.04), a marker associated with SC proliferation, remained similar between the two SC populations. Taken together, the activation of Notch signaling and the increased expression of *MYOD1* and *MYOG* likely contribute to the enhanced proliferation and differentiation capabilities of fast-growing satellite cells.

## 4 Discussion

Over the past several decades, selective breeding for accelerated growth performance in poultry has produced faster-growing lines with enhanced breast muscle development compared to non-selected slow-growing lines (Havenstein et al., 2007). Growth-selected turkeys exhibit increased myofiber diameter and reduced connective tissue spacing in the p.major muscle compared to non-selected counterparts (Velleman et al., 2003). However, alongside this selection, reports of undesirable muscle fiber damages, such as deep pectoral myopathy and focal myopathy (Siller, 1985; Wilson et al., 1990), have emerged. These observed alterations in p.major muscle development, growth trajectory and morphological characteristics possibly correlate with changes in the SC populations intrinsic to the p.major muscle (Ferreira et al., 2020; Pejskova et al., 2024). Indeed, previous comparative investigations between faster-growing Nicholas Commercial (NC) turkeys and random-bred populations demonstrated that SCs derived from NC turkeys exhibit enhanced proliferative and differentiation capacities, elevated intracellular lipid content, and increased susceptibility to thermal stress (Xu et al., 2021a; Xu et al., 2021b).

Satellite cells are not a homogenous population of cells (Feldman and Stockdale, 1991; Kuang et al., 2007; Ono et al., 2010). McFarland et al. (1995) identified substantial intrinsic heterogeneity among SC populations derived from the same p.major muscle, demonstrating variability in proliferative and differentiative rates as well as differential growth factor responsiveness. Building on the SCs isolated in McFarland et al. (1995) study, the current research investigated the transcriptomic differences between the SC populations with different proliferation rate. The results indicated substantial transcriptomic differences between the 2 cell populations. Specifically, over 5,000 genes exhibited differential expression, with 2,675 genes upregulated in late clones and 2,672 genes upregulated in early clones. Gene ontology analysis from two independent sources highlighted substantial differences. Pathways prevalent in early clones were primarily associated with the establishment of fundamental muscle structure and cytoskeletal development. Conversely, the functional analysis of late clones revealed pathways involved in extracellular receptor interactions, cell communication, signaling, and cytokine activity. These functional annotations suggest that SCs exhibit considerable functional diversity. The maintenance of normal muscle homeostasis depends on a balance between these distinct SC populations. The current study is the first to genetically characterize two distinct SC populations from the same turkey p*.* major muscle.

Nutrient availability influences SC activity, which in turn affects muscle development and structural changes. Studies have demonstrated that reduced nutrient availability diminishes SC proliferation and differentiation, resulting in lower body weight and *p. major* muscle mass (Halevy et al., 2000; Mozdziak et al., 2002; Halevy et al., 2003; Powell et al., 2013; Harthan et al., 2014). Furthermore, the *p. major* muscles from chicks subjected to feed restriction during the first week post-hatch exhibit increased muscle fiber necrosis and adipose deposition, along with altered expression of myogenic transcriptional regulatory factors that control SC proliferation and differentiation (Velleman et al., 2010; Velleman et al., 2014a). Interestingly, if nutrient restriction is initiated in the second week post-hatch, these adverse effects on myogenic gene expression, muscle fat content, and morphological structure are not observed (Velleman et al., 2014a; Velleman et al., 2014b). These findings suggested that nutrition affects SC biology and that this effect is time sensitive. Therefore, a future direction of the current study is to investigate whether nutrition could affect early and late clones differently. While the current studies have genetically characterized two populations of SCs, we believe there are additional distinct populations of SCs, as suggested by numerous publications from human biomedical research (Dell'Orso et al., 2019; Barruet et al., 2020; Sousa-Victor et al., 2022). Consequently, it is crucial to identify and characterize more SC populations in turkey *p. major* muscles. A further step is to explore whether specific nutritional supplements can enhance the growth of particular SC populations. Moreover, it is important to assess the overall contribution of SC heterogeneity to muscle growth and development. By understanding these dynamics, we can potentially develop nutritional strategies to optimize muscle growth in livestock, thereby improving meat quality and production efficiency.

The Organization for Economic Co-operation and Development (OECD) and the Food and Agricultural Organization (FAO) of the United Nations, (2023) predicts that the global population will grow by 11% from 7.9 billion in 2022 to 8.6 billion in 2032. Concurrently, global meat production is projected to increase by 15% by 2032, with poultry meat anticipated to represent 48% of this growth over the next decade (OECD/FAO, 2023). This underscores the poultry industry’s need to enhance animal growth and meat production. Given the critical role of SCs in driving muscle growth, future nutrition and breeding strategies should incorporate considerations of SC dynamics to optimize these processes effectively.

## 5 Conclusion

This study has identified specific genes and gene pathways that differentiate fast-growing and slow-growing satellite cells, isolated from single-cell colony expansions. Our RNA-seq analysis offers a snapshot of gene expression changes along a continuum, which may correspond to functional variations in gene products. The transcriptomic profiles of early and late clones show significant differences, associated with distinct functional annotations. We hypothesize that normal muscle function and homeostasis are sustained by delicate balances among different SC populations. Disruption of this equilibrium could hamper muscle growth and may lead to reduced meat production. Further research is needed to definitively determine how SC heterogeneity contributes to the overall muscle growth.

## Data Availability

The data presented in the study are deposited in BioProject repository, accession number PRJNA1196520. The data has released to public and can be accessed at https://www.ncbi.nlm.nih.gov/bioproject/PRJNA1196520.
