# Supplementary figures and images for: Molecular characterization of the heterogeneity of satellite cell populations isolated from an individual Turkey pectoralis major muscle
Source: Front Physiol. 2025 Feb 20;16:1547188. doi: 10.3389/fphys.2025.1547188 (PMC11882874; doi:10.3389/fphys.2025.1547188)

**A** GO: 0031012  
extracellular matrix

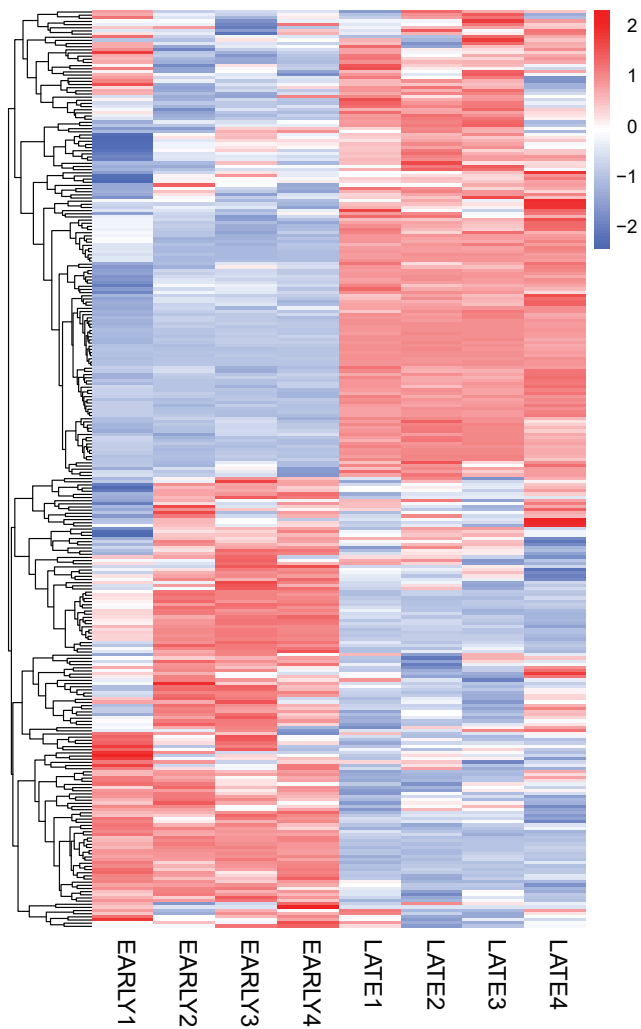

**B** GO: 0030546  
signaling receptor activator activity

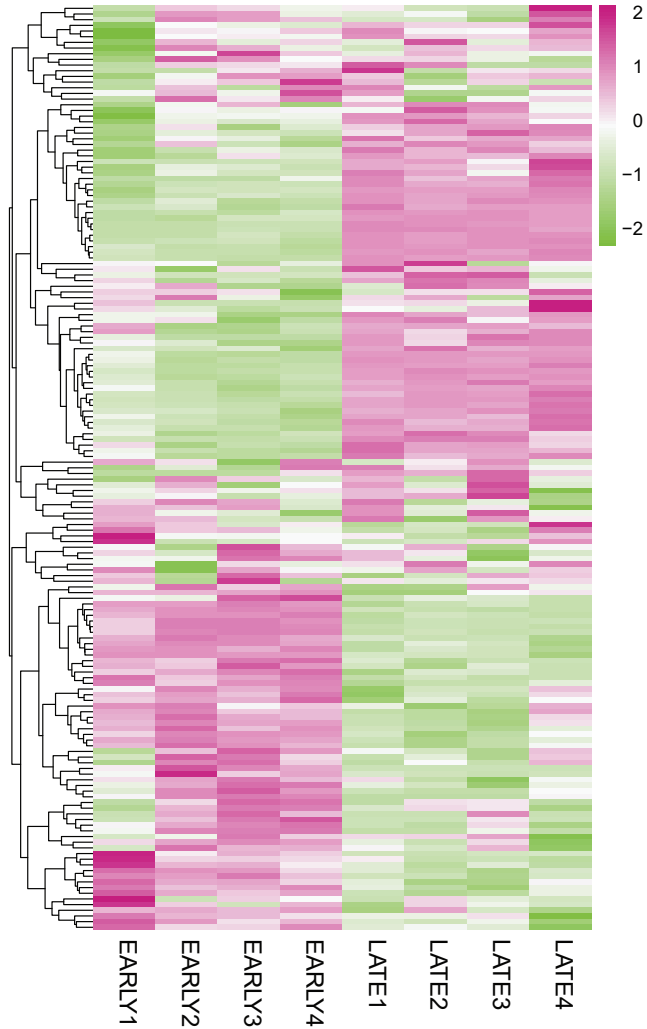

Supplement: Supplementary file 1 [file DataSheet2.pdf]

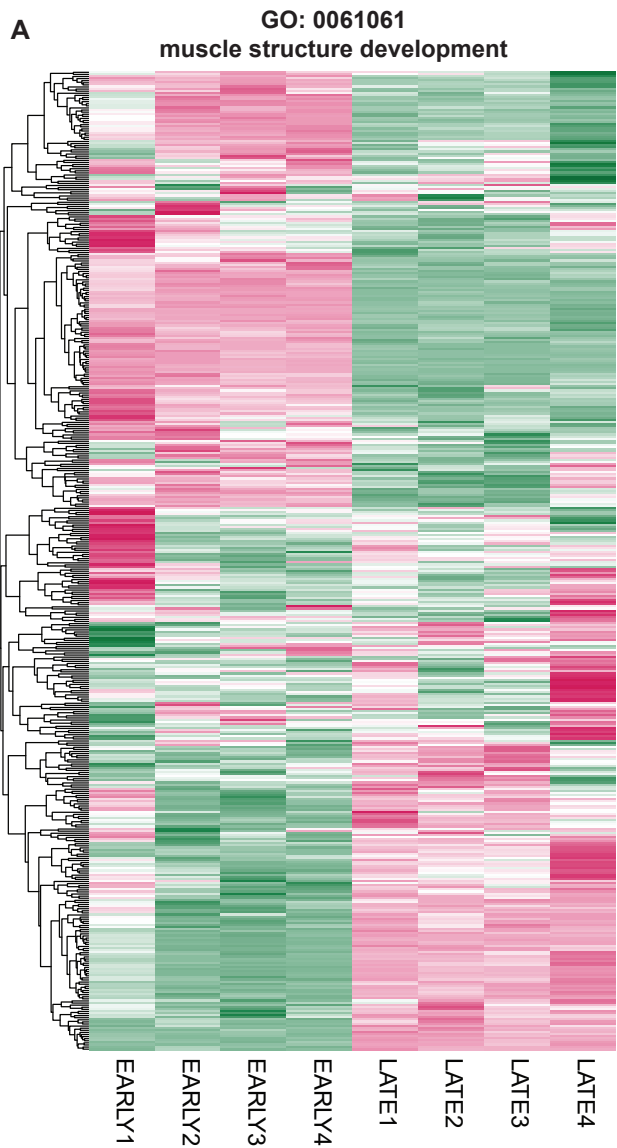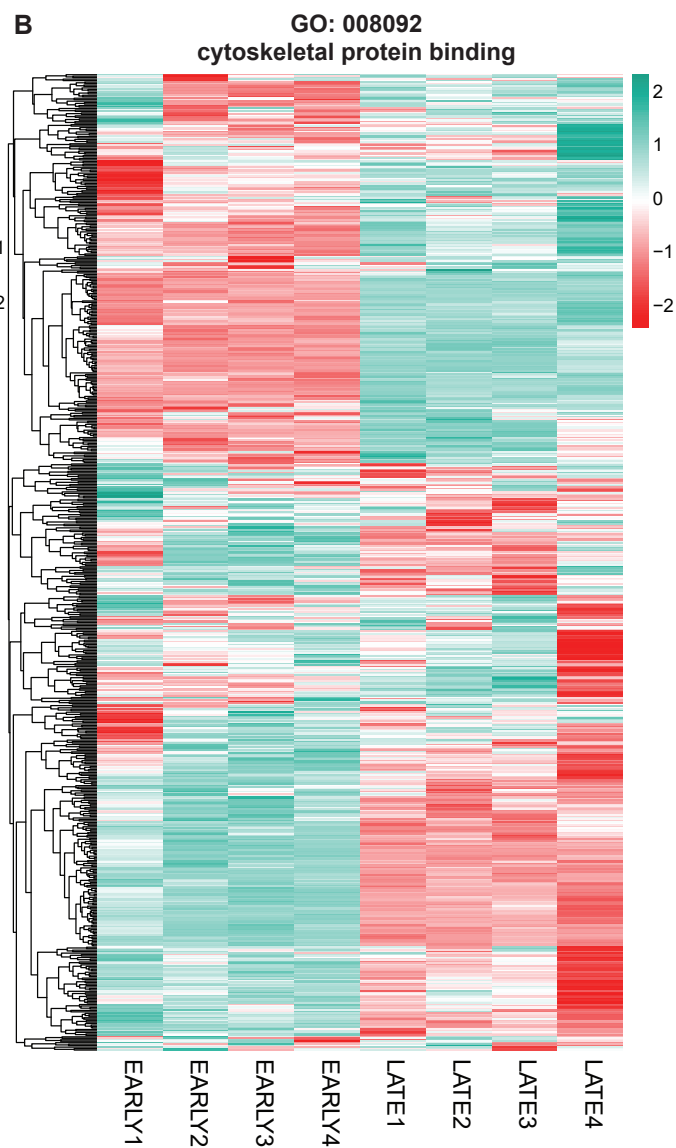

Supplement: Supplementary file 2 [file DataSheet1.pdf]
